# Nature of Carbon Black Reinforcement of Rubber: Perspective on the Original Polymer Nanocomposite

**DOI:** 10.3390/polym13040538

**Published:** 2021-02-12

**Authors:** Christopher G. Robertson, Ned J. Hardman

**Affiliations:** 1 Endurica LLC., Findlay, OH 45840, USA; cgrobertson@endurica.com; 2 Monolith Materials, Monolith Technical Center, Lincoln, NE 68522, USA

**Keywords:** carbon black, elastomers, hyperelastic material, stress–strain behavior, polymer–filler interactions, reinforcement

## Abstract

Adding carbon black (CB) particles to elastomeric polymers is essential to the successful industrial use of rubber in many applications, and the mechanical reinforcing effect of CB in rubber has been studied for nearly 100 years. Despite these many decades of investigations, the origin of stiffness enhancement of elastomers from incorporating nanometer-scale CB particles is still debated. It is not universally accepted whether the interactions between polymer chains and CB surfaces are purely physical adsorption or whether some polymer–particle chemical bonds are also introduced in the process of mixing and curing the CB-filled rubber compounds. We review key experimental observations of rubber reinforced with CB, including the finding that heat treatment of CB can greatly reduce the filler reinforcement effect in rubber. The details of the particle morphology and surface chemistry are described to give insights into the nature of the CB–elastomer interfaces. This is followed by a discussion of rubber processing effects, the influence of CB on crosslinking, and various chemical modification approaches that have been employed to improve polymer–filler interactions and reinforcement. Finally, we contrast various models that have been proposed for rationalizing the CB reinforcement of elastomers.

## 1. Introduction

The term reinforcement can have a variety of meanings when considering the effects of adding nanometer-scale particles such as carbon black (CB) to an elastomer. Without fillers, crosslinked rubbers are generally weak materials. Reinforcing filler particles enhance the tensile strength, tear resistance, and abrasion resistance of rubber—to varying degrees based on the particle size and shape features and nature of elastomer–filler interactions—while also increasing static and dynamic stiffness characteristics. All these mechanical property improvements can be considered reinforcement. Our focus in this review is on the influence of CB on the tensile stress–strain curve, in particular the stress upturn (strain stiffening) behavior in the medium to large strain regime.

**Figure 1:**
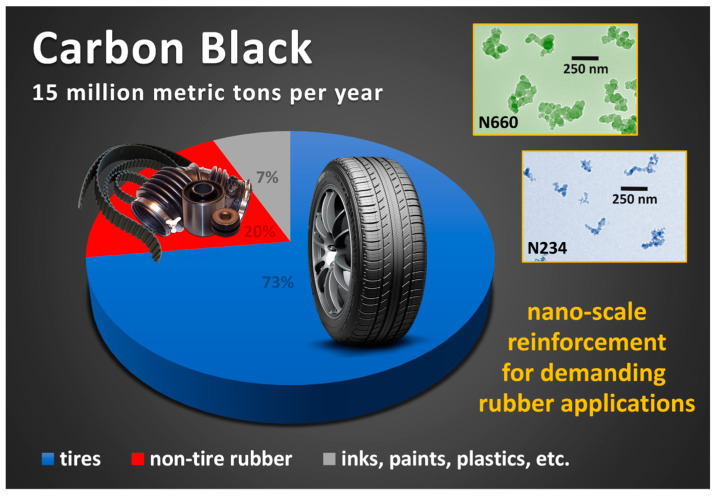
Global annual production of carbon black and its distribution in the indicated end-use industrial application areas, based on reported data [5]. Representative examples of carbon black (CB) aggregates are shown in the transmission electron microscopy (TEM) images for N234 and N660 types to show the nano-scale morphologies that are key to reinforcement.

The most prevalent filler type in demanding rubber applications such as automobile tires and mechanical rubber goods is carbon black, which has many grades encompassing a large range of particle sizes (surface areas) and nano-structured aggregate morphologies for producing different property balances. There is ongoing research interest in employing other carbon allotropes (e.g., graphene, carbon nanotubes) for reinforcing elastomers [1,2,3], and the use of precipitated silica in rubber applications is growing [4]. However, carbon black is by far the dominant filler in rubber products. The global production of carbon black is 15 million metric tons per year, and 93% of this material goes into rubber applications (automobile tires (73%) and non-tire rubber products (20%)), with the remaining 7% used in paints, coatings, inks, and plastics compounding (Figure 1) [5]. This industrial importance of carbon black provides motivation for our review on the origin of the mechanical performance enhancements provided by carbon black in rubber compounds used in these challenging applications.

Scientific literature on CB reinforcement of rubber has been steadily amassing since the early 20th century, producing over 10,000 publications including nearly 100 papers with “carbon black”, “reinforcement”, and “rubber” in the article title. This sea of literature makes it a challenge to gain a conclusive scientific understanding of why carbon black is so successful at giving high degrees of reinforcement in industrial rubber applications. Toward gaining a clearer picture, we will first review some key experimental observations. This set of phenomenological facts provides a consistency check for any proposed rationalization of CB-induced stiffness and strength increases in rubber, including the various theories that have been offered.

**Figure 2:**
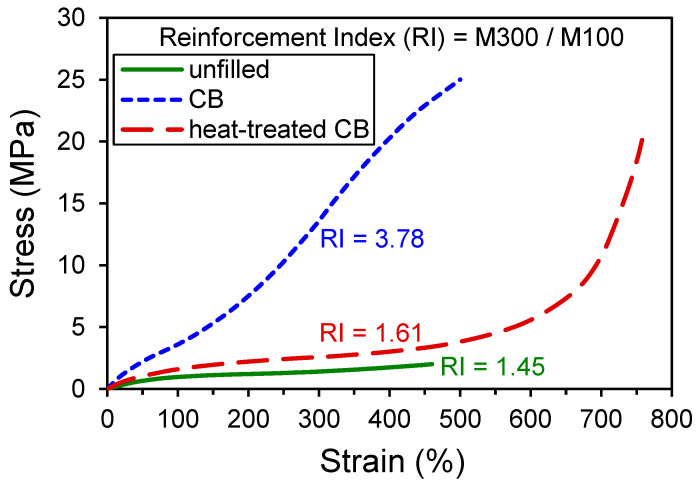
Tensile stress–strain results for non-crystallizing rubber vulcanizates including unfilled rubber and rubber compounds filled with CB and heat-treated CB. Data replotted from Medalia and Kraus [6].

Perhaps the simplest way to look at the influence of CB on the mechanical behavior of elastomers is to compare unfilled and filled rubber in the standard tensile stress–strain test conducted at room temperature. Examples of such stress–strain curves from Medalia and Kraus [6] are reproduced in Figure 2, wherein the large impact of CB on the response can be noted immediately. One measure of filler influence on the stress–strain curve is to compare values of reinforcement index (RI) with and without filler, where RI = M300/M100. This ratio of the stress at 300% strain (M300) to the stress at 100% strain (M100) has a value of 3.78 for the CB-filled material shown in Figure 2 which is significantly greater than RI = 1.45 for the unfilled rubber. If the carbon black particles are heat treated, then the RI of the resulting rubber composite is only slightly higher (RI = 1.61) than the unfilled material. The remarkable extent of reinforcement from CB can be nearly extinguished by high temperature annealing of the filler.

Brennan, Jermyn, and Boonstra [7] showed how reinforcement and other rubber properties were affected when comparing heat-treated carbon blacks with their untreated CB counterparts. They heat treated two grades of CB at 3000 °C for one hour, and a summary of results in styrene–butadiene rubber (SBR) formulations is given in Table 1. The reductions in tensile properties (M300, tensile strength, etc.) for heat-treated CB compared to untreated reference CB are consistent with the results in Figure 2 already discussed. A substantially lower bound rubber and 2 to 3 times faster abrasion rate were also noted for the heat-treated fillers compared to normal carbon blacks. These researchers determined bound rubber from a three-day extraction experiment using toluene solvent. Bound rubber is the portion of the elastomer that is strongly interacting with the filler such that it cannot be removed from the uncured compound by solvent extraction. This highlights the importance of the polymer–filler interface/interphase to reinforcement.

**Figure 3:**
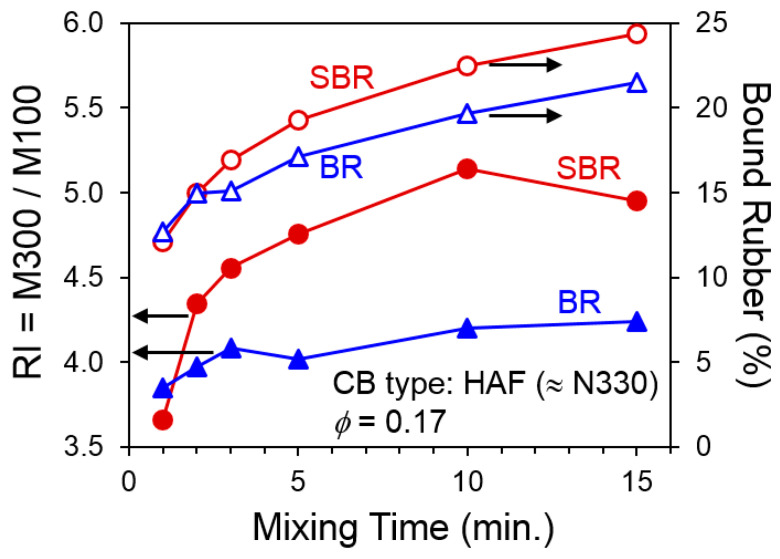
Reinforcement index and bound rubber versus mixing time for CB-filled SBR and BR compounds. Data replotted from Brennan, Jermyn, and Boonstra [7].

Another important aspect of the influence of CB filler on mechanical behavior is the observation that reinforcement index—steepness of the stress–strain curve—typically increases for longer mixing times. This is illustrated in Figure 3 using results from Brennan et al. [7] for CB-filled butadiene rubber (BR) and SBR materials, with the magnitude found to be larger for SBR in this particular study. Bound rubber also rises with increasing compounding time for both compounds. 

**Figure 4:**
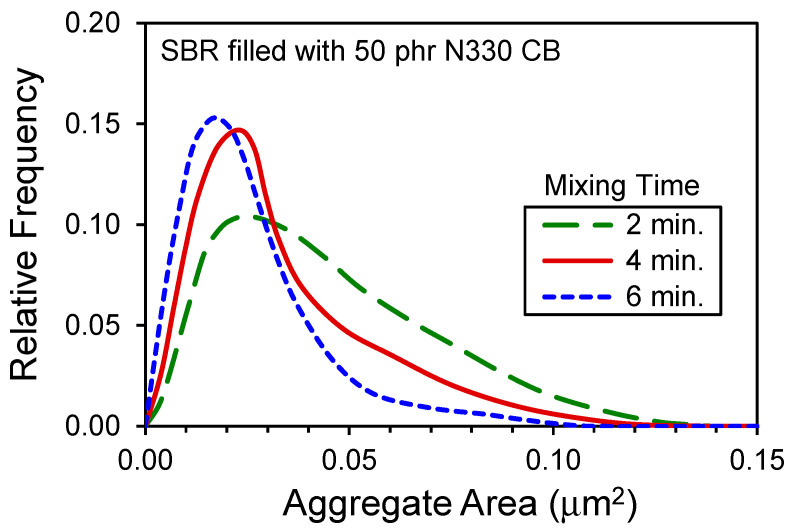
CB aggregate distributions from analysis of TEM images that show progressive aggregate breakage during mixing. Data replotted from Klüppel [8].

The rubber mixing process also affects the morphology of the carbon black aggregates. Carbon black is composed of primary particles that are fused into larger-scale, complex-shaped aggregates during the CB manufacturing process. Once the CB pellets/granules and then agglomerates are sequentially broken down and dispersed in the internal mixer, the aggregates are then exposed to the shearing forces and can fracture. Detailed analysis of transmission electron microscope (TEM) images from microtomed specimens from the cured rubber can allow aggregate size distributions to be characterized for investigating aggregate breakage. Some typical results published by Klüppel [8] are given in Figure 4 which show the shifting of the aggregate distribution to smaller sizes due to this breakage. 

**Figure 5:**
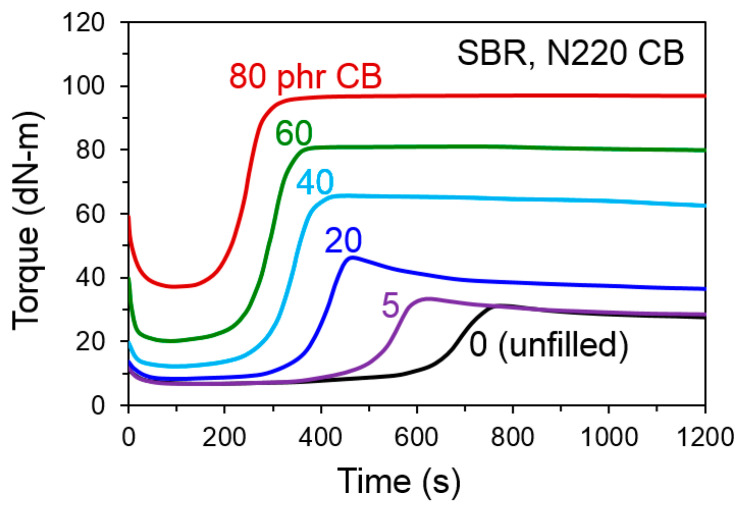
Rheological cure curves at 160 °C for sulfur vulcanization of SBR filled with N220 CB at the indicated loadings. Data replotted from Hossein and Razzaghi-Kashani [9].

When CB is introduced into rubber, the overall sulfur cure chemistry speeds up [9,10], and this is an important aspect that must be considered in any attempt to rationalize carbon black reinforcement effects. Depending on the oil feedstock characteristics, commercial grades of CB used in the tire and rubber industry have sulfur contents that typically range from 0.2 to 2 wt.%. However, this is not free sulfur but rather is chemically bound to the CB as will be discussed later. Increasing CB concentration in a rubber formulation systematically moves the cure curves to shorter times, as shown in Figure 5 using data from a study by Hosseini and Razzaghi-Kashani [9], and a very significant acceleration is noted with only 5 phr of CB. If CB catalyzes vulcanization or adsorbs curatives during mixing, it is likely that the crosslink density is higher near the filler surfaces compared to the bulk rubber. It is possible that chemical linkages are formed between elastomer chains and functional groups on CB surfaces during vulcanization. Bound rubber testing on uncured rubber gives some insights into elastomer–filler interactions. However, it is the nature of the interfacial region in the final cured rubber—as possibly impacted by any CB surface effects on the sulfur vulcanization process and its spatial distribution—that influences the extent of reinforcement in the rubber.

## 2. Regions of Reinforcement

**Figure 6:**
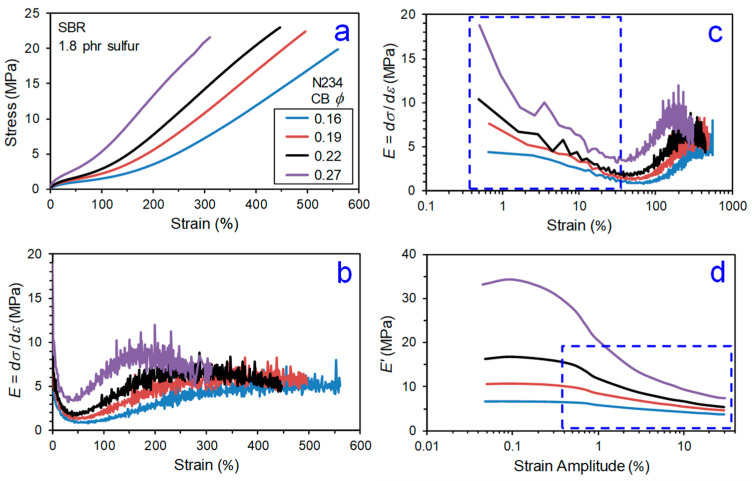
Effect of CB loading on tensile stress versus strain behavior at 23 °C (**a**) and its derivative (slope; modulus (*E*)) for CB-filled SBR plotted versus linear strain (**b**) and logarithmic strain (**c**). Unitless strain (not %) used when determining *E*. Also shown is strain amplitude dependence of dynamic storage modulus (*E’*) at 60 °C and 10 Hz (**d**). Each blue dashed-line box has the same dimensions in both (**c**) and (**d**) to allow comparisons of the strain-induced softening (Payne effect). Results are replotted from Warasitthinon and Robertson [11].

**Figure 7:**
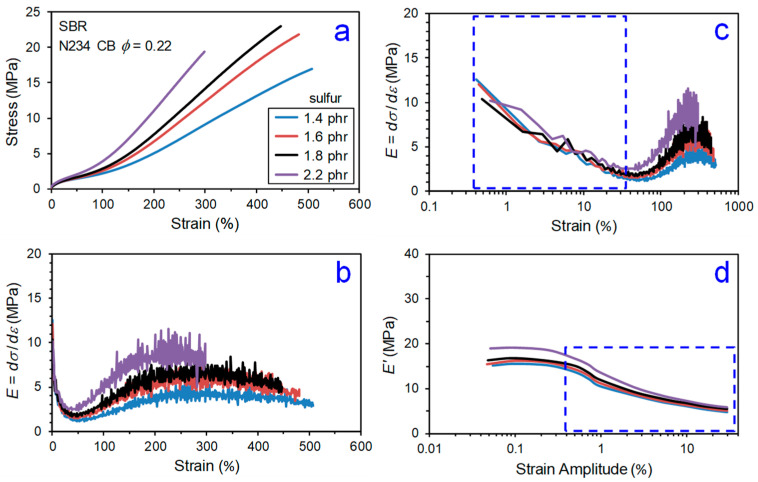
Effect of sulfur loading on tensile stress versus strain behavior at 23 °C (**a**) and its derivative (slope; modulus (*E*)) for CB-filled SBR plotted versus linear strain (**b**) and logarithmic strain (**c).** Unitless strain (not %) used when determining *E*. Also shown is strain amplitude dependence of dynamic storage modulus (*E’*) at 60 °C and 10 Hz (**d**). Each blue dashed-line box has the same dimensions in both (**c**) and (**d**) to allow comparisons of the strain-induced softening (Payne effect). Results are replotted from Warasitthinon and Robertson [11]. Accelerator to sulfur ratio was maintained constant in this study.

**Figure 8:**
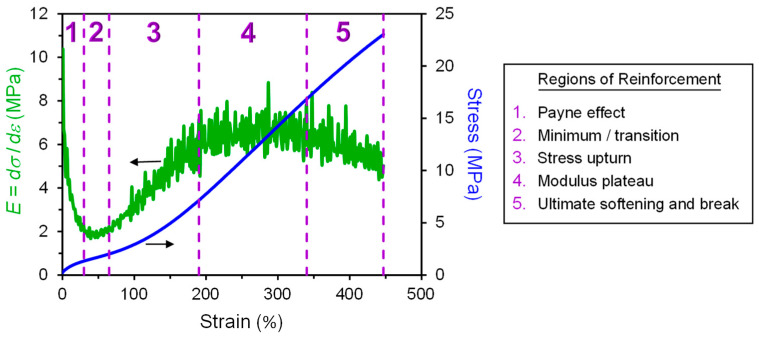
Regions of filler reinforcement illustrated using tensile stress–strain curve and its derivative (slope; modulus (*E*)) for N234 CB-filled SBR (*ϕ* = 0.22) with 1.8 phr sulfur. Stress–strain data from Warasitthinon and Robertson [11]. Unitless strain (not %) used when determining *E*. The dashed vertical lines separate the five regions.

The mechanical behavior of CB–elastomer composites is complex. Even a basic tensile stress–strain experiment involving stretching a test specimen of particle-reinforced rubber in simple tension at a fixed strain rate until rupture has several regions to consider in discerning the impact of CB on reinforcement. Previously published results [11] on SBR-based model tire tread compounds filled with N234 CB are used to illustrate this in Figure 6, Figure 7 and Figure 8. In Figure 8, we identify five regions of reinforcement in the stress–strain response:

1. Payne effect,
2. Minimum/transition,
3. Stress upturn,
4. Modulus plateau, and
5. Ultimate softening and break.

These zones are defined based on the slope of the stress–strain response, which is the modulus, *E* = dσ/dε, wherein strain is unitless (not %) in the determination of the derivative.

Region 1 is where the Payne effect is observed, and this is best noted using a logarithmic strain axis (e.g., Figure 6c). The Payne effect [12,13,14,15,16,17] is a well-known dynamic strain-softening phenomenon in particle-filled rubber, and an early documentation of this behavior was provided in 1944 by Dillon, Prettyman, and Hall [18]. A significant reduction in dynamic storage modulus and appearance of a peak in loss tangent (tanδ) as the oscillatory strain amplitude is increased are characteristics features of the Payne effect, with most of the hysteretic softening occurring in the 0.1 to 10% dynamic strain amplitude range. The presence of a strain-sensitive filler network of percolated particle–particle contacts is predominantly responsible for the Payne effect [15,19,20,21,22], with polymer dynamics at the polymer–filler interfaces also contributing to a lesser extent [20,23,24,25]. This behavior is not exclusive to dynamic mechanical response, as the filler network break-up also controls the small-strain part of a constant strain rate tensile test. Comparing Figure 6c with Figure 6d and Figure 7c with Figure 7d confirms that the first part of the tensile test (Region 1) is exhibiting the Payne effect. This is why special constitutive modeling efforts are required to capture this small-strain part of the stress–strain behavior for highly filled compounds which have very significant filler networking [26]. This reinforcement region is greatly influenced by CB loading (Figure 6) but not significantly affected by crosslink density (Figure 7).

The modulus goes up in Region 3 as strain is increased, which is opposite to the Payne effect strain softening in Region 1. This stress upturn (strain stiffening) is influenced by finite extensibility of polymer network chains [27,28,29] and strain-induced crystallization for some stereoregular elastomers such as natural rubber [30,31]. Carbon black affects both of these phenomena [32,33], but we will focus on the universal polymer network part in this review. Increasing sulfur level, and hence density of crosslinks, has a similar effect to increasing the CB concentration (Figure 6 and Figure 7) on the Region 3 stress upturn behavior, making is challenging to sort out CB reinforcement features given the previously mentioned impact of CB on the crosslinking process.

The intersection of Region 1 strain softening and Region 3 strain stiffening yields a finite transition zone (Region 2) where *E* is at a minimum, *E_min_*. Widespread in the literature are comparisons of experimentally measured modulus values of particle-filled rubber to predictions of basic hydrodynamic models (Einstein [34], Guth [35], and others [36,37]), with filler volume fraction, *ϕ*, as the key material parameter. Such evaluations are really only meaningful in this quite narrow Region 2 window, as filler networking dominates at lower strains and complex filler effects on the stress upturn govern at higher strains.

**Figure 9:**
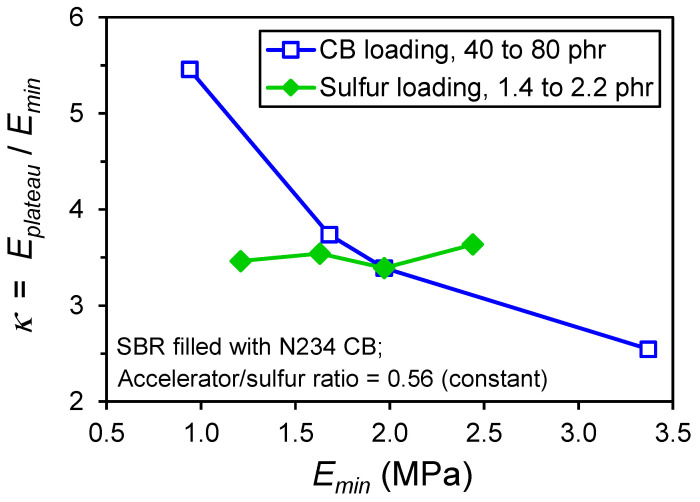
Robertson–Hardman reinforcement index *κ* determined from the literature data [11] shown in Figure 6 and Figure 7.

After the stress upturn regime is a zone (Region 4) where the modulus is constant at a plateau value, *E_plateau_*. We propose a reinforcement index, *κ* = *E_plateau_*/*E_min_*, as a better indicator of the reinforcement capability of particles compared to the common RI = M300/M100. The strain range where the stress upturn in Region 3 occurs depends on crosslink density and filler loading (see Figure 6 and Figure 7), such that the 100% and 300% points on the tensile curve are in different places for different compounds. Some rubber compounds with higher crosslink densities or higher filler loadings have an ultimate elongation less than 300%, which precludes the use of the traditional reinforcement index, RI. The *κ* index is more fundamentally identified from the stress–strain response and thus universally applicable. The values of the strain boundaries for the various regions illustrated in Figure 8 are additional parameters that can be utilized for contrasting reinforcement effects in different elastomer formulations. This new reinforcement factor concept was applied to the data shown in Figure 6 and Figure 7, yielding the *κ* results in Figure 9. Distinct trends are noted for the dependences of *κ* on CB loading versus sulfur level. The *κ* is invariant to crosslink density but shows a significant decrease with increasing carbon black loading, suggesting that this index is sensitive to filler reinforcement effects in elastomers. 

Ultimate softening and then fracture take place in the final zone (Region 5). The modulus slowly decreases due to breaking of some crosslinks and polymer chains at the higher strains and growth of microcracks around crack precursors in the rubber. In particle-filled rubber, this softening also includes polymer–particle slippage, dewetting, and vacuole formation that progressively occur as strain is increased [6,38,39]. The final tensile strength of the material is dictated by tear strength and crack precursor size [40].

## 3. Structural and Chemical Characteristics of Carbon Black

In a 1957 paper, Watson referred to the carbon black reinforcement of elastomers and stated, “Some workers believe that the mechanism is at least partly chemical, whereas others claim that it is entirely physical in nature” [41]. The same statement can be made today, 64 years later. Carbon black is an exceedingly nuanced material and is only now being fully understood for the full depth of its structural and chemical complexity. Further, when speaking of reinforcement, we are referring to the effects of CB on physical properties that are measured on the final cured rubber material. Sulfur vulcanization of rubber is a complex multi-step chemical process [42,43] that can be potentially affected by the presence of CB, as will be considered later in this review.

Elementally, carbon black from the furnace process is at least 92% carbon. Given current pyrolysis fuel oil feedstock in North America, the sulfur content ranges from 0.6 to 1.5% depending on the specific feedstock and the grade of carbon black. The other main elements are oxygen, nitrogen, and hydrogen, as can be seen in Table 2 for N234 and N660 grades.

Ash in furnace grade carbon black is typically in the range of 0.2 to 0.8% and is normally dependent upon the purity of the quench and pelletization water as well as the quality of the feedstock. Six elements comprise 90+% of the ash in furnace carbon black; Al, Ca, Mg, K, Si, and Na. For extremely pure grades of carbon black with less than 0.03% ash, the materials of construction of the reactor, the backend processing, and even the silos that store the carbon black can make a meaningful difference. The last item at the carbon black surface is the group of polycyclic aromatic hydrocarbons (PAH) such as pyrene, naphthalene, and anthracene which for typical furnace CB is in the range of several hundred ppm [44].

**Figure 10:**
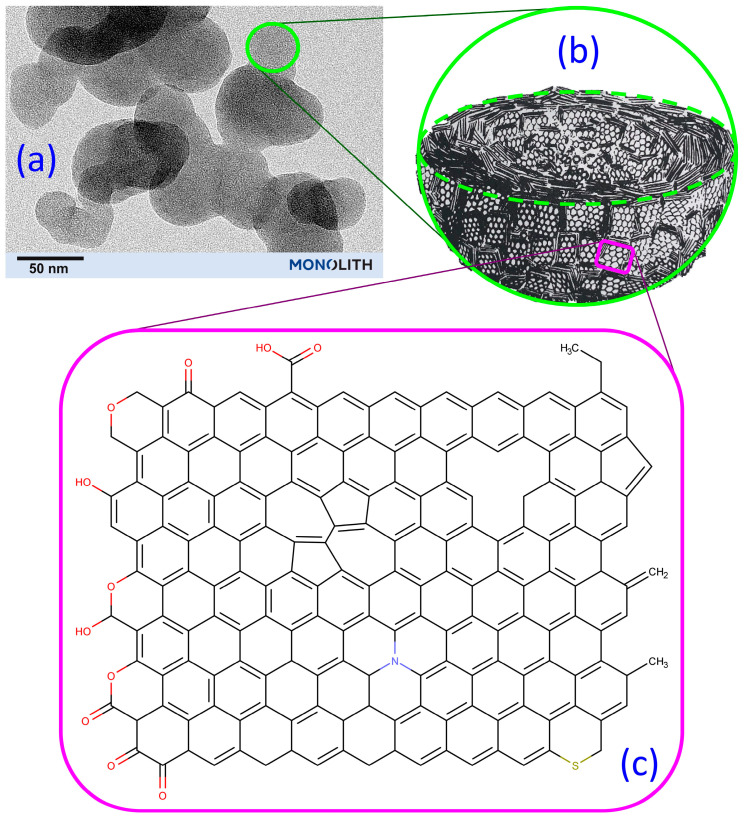
Different levels of structure and chemistry in carbon black: (**a**) TEM image of N660 carbon black at the indicated magnification; (**b**) schematic of a primary particle (cut in half to show the internal structure) that is composed of stacks of graphene sheets (adapted from Heidenreich et al. [48]); and (**c**) depiction of the different chemical groups on a graphene sheet in CB along with possible defects in the sheet. The various functional groups and defects are described in the discussion text.

The structural and chemical features of carbon black are illustrated in Figure 10. The main three characteristics of carbon black are the surface area, structure, and surface chemistry. The surface area of carbon black is a measure of the primary particle size and is evaluated by a variety of adsorption methods [45,46]. The methods include gas to solid adsorption where gasses such as nitrogen or carbon dioxide are adsorbed out of the gas phase and then surface area is calculated using the BET equation. Nitrogen surface area (NSA) is accordingly a common parameter used to characterize CB surface area. The second main method to measure surface area is to use a solute at a known concentration that forms a monolayer on the surface of carbon black such as iodine or cetyl trimethyl ammonium bromide (CTAB). In the case of CTAB, the statistical thickness surface area (STSA) is then calculated by performing a titration on the remaining solute that has not adsorbed to the carbon black surface. The same is true for iodine, however, iodine is a small enough molecule that not just the external surface area (STSA), but all of the surface area is measured including the porosity. The types of furnace carbon black that are used to reinforce rubber have a broad range of surface areas from 31 to 140 m^2^/g, as can be seen in Table 3 [47]. 

**Figure 11:**
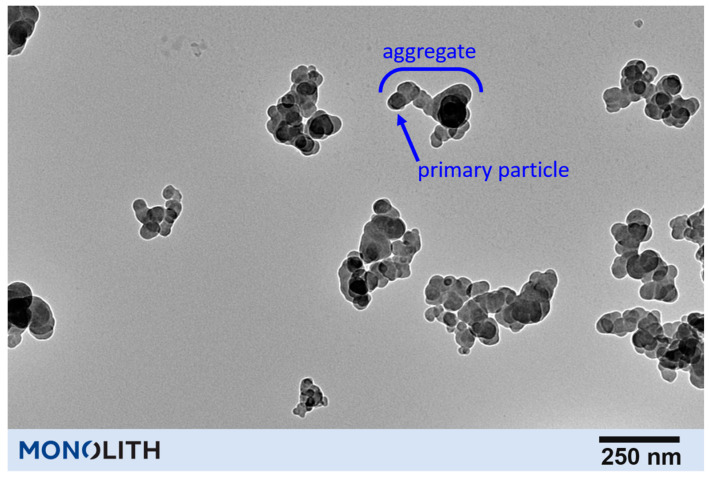
TEM image of N660 carbon black that shows the aggregates that are composed of smaller primary particles.

The size of the primary particles controls the surface area, but the shape/structure of the aggregates is also important. Figure 11 is a transmission electron microscopy (TEM) image of N660 carbon black that shows the aggregates that are composed of smaller primary particles. This structure of carbon black is quantified using the oil absorption number (OAN). The OAN is a measure of the number of primary particles that are fused together and more specifically the amount of intra-aggregate void volume of the nano-structured aggregates. The OAN is measured through the dripping of oil at a constant rate into a cylinder containing carbon black. The cylinder contains the carbon black and two rotors that are moving at constant speed. The torque required to turn the rotors reaches a maximum where the entirety of the void volume between the aggregates has been filled. The curve is fitted to a quadratic and a pre-determined method of calculating the OAN value is used to give the official OAN or structure value. Compressed OAN or COAN uses the same absorption method, however, the carbon black pellets are submitted to extreme pressure in a piston four times prior to the measurement. In this way the pellets are compressed or crushed in a way that is supposed to mimic the crushing or shearing in the rubber processing equipment. Another method for measuring the mesoporosity or intra-aggregate volume is to perform mercury porosimetry which can provide more detail than the typical OAN test. For a complete description of the physical parameters and testing please, see the following reference [44].

While the first two characteristics of carbon black are fairly straightforward, the third parameter—surface chemistry or surface energy—is more complex and consequently subject to thorough debate. If one specifies surface chemistry, then it is implied that specific chemical groups are being measured such as Boehm titrations for specific oxygen functionality at the surface [49,50]. Electron spin resonance (ESR) and X-ray photoelectron spectroscopy (XPS) are other methods that would typically be used to measure the surface chemistry. For surface energy, common measurements are inverse gas phase chromatography (IGC) and ethylene adsorption [51,52]. In the remaining paragraphs of this section, we will discuss some items that affect this third descriptor of carbon black.

Carbon black, as can be seen in Figure 10b, is mostly carbon arranged in small stacks of graphene sheets. A crystal within carbon black can be envisaged as a cube approximately 1.5 × 1.5 × 1.5 nm (slight function of CB type but not as much as primary particle size). For simplicity, the reader can picture 4–5 sheets of graphene on top of each other for the typical crystallite size of graphene in carbon black. The length scale of these crystallites in the stacking direction is L_c_. A primary particle can be thought of as hundreds or thousands of these cubes packed together in such a way as to make a spherical ball. The interior of this ball is known to be more amorphous and the exterior is known to be more graphitic [44]. 

Carbon black has been described as quasi-crystalline. In typical graphite, the graphene sheets are stacked in such a way that there is A-B-A-B repeated stacking like a very ordered deck of cards. In carbon black, the stacking is characterized as turbostratic, which means that the graphene sheets are not aligned so as to allow the lowest energy overlap between the pi orbitals of the intervening layers, and this causes the interlayer spacing (d_002_) to change from the value found in graphite of 0.334 nm to values for carbon black in the 0.35 to 0.36 nm range. The most likely reason for the turbostratic structure is that the manufacturing process for furnace carbon black involves heating oil feedstock in an oxygen-limited atmosphere to temperatures in excess of 1600 °C and then immediately quenching the process fractions of a second after it has started. This kinetically freezes the layers in place in a disordered fashion. Crystallinity of carbon black can be measured by powder X-ray diffraction or Raman spectroscopy.

Figure 10c illustrates the variety of chemical groups and defect type structures that are at the surface of carbon black. For reference, this graphene sheet is approximately 2.0 × 2.5 nm which is slightly larger than the crystalline domain as measured by X-ray diffraction for furnace CB. It has long been known, and it was predicted theoretically before it was proven, that 5 membered rings are present during the formation of carbon black [53]. Stone–Wales defects are 5 and 7 membered rings (see center of Figure 10c), and these are well known in graphene and almost certainly present in carbon black [54]. Additionally, hole defects are well known as well as other types of defects that are present in the surface structure of most if not all allotropes of carbon [55]. These defects become readily apparent when studying graphene and nanotubes due to the advent of aberration-corrected TEM and similar technologies.

The possible types of carbon black surface functional groups are also shown in Figure 10c. The top left-hand side of the depiction starts with a carboxylic acid and then moving counter-clockwise there is a carbonyl, an ether, a phenol, a lactol, an ester, and ending with a quinone at the bottom left side of the graphene sheet. These are the typical oxygen groups that are depicted as surface chemistry of carbon black. Other types of surface chemistry that are prevalent in the graphene literature are Stone Wales defects, heteroatom substitution (N, S), site vacancies or holes (upper right) as well as potential vinyl, allylic, and methyl groups. Typically, it is only oxygen that is displayed as a heteroatom in normal drawings of the graphene sheets, but the sulfur and nitrogen atoms could introduce interesting characteristics, even in very low quantities. The sulfur content of CB does not change significantly after heat treatment at temperatures from 900 to 1200 °C (Table 2). These temperatures are well above the boiling point of sulfur (445 °C), which indicates that the sulfur is chemically bound as depicted in Figure 10c. On the far right-hand side of Figure 10c, a lone pendant 5 membered ring is present. Additionally, in the bottom center one can observe the presence of unconjugated double bonds that lack aromatic character and are therefore more reactive. The unconjugated double bonds that are present in carbon black also can contribute to chemical reactivity as a Diels–Alder reactant with other dienes and dienophiles [56]. Researchers have also reacted benzoyl peroxide through laser illumination and found that the benzene radical reacts with graphene and double layer graphene to form a single bond to the graphene surface [57].

The functional groups on the edges of the graphene sheets on the CB surface are reduced/eliminated when carbon black is annealed at high temperatures. Our elemental analysis results summarized in Table 2 show that increasing the heat treatment temperature from 900 to 1200 °C resulted in systematic and substantial reductions in the oxygen and hydrogen concentrations for N234 CB. Heat treatment of N660 CB was also performed at a single annealing temperature of 1000 °C with the same effects observed. Nitrogen content for these carbon blacks was also substantially decreased with heat treatment whereas sulfur was largely unchanged. We emphasize that these changes to the CB surface functionality from heat treatment occur without significant changes to surface area or to the measured crystalline parameters, L_c_ and d_002_, that are also reported in Table 2. The term graphitization is often used to describe heat treatment of CB, and extreme heat treatment in the 2000–3000 °C range can allow for growth and perfection of the graphitic structure. The annealing temperatures for removing oxygen-containing functional groups from the carbon black surface are much lower (900 to 1200 °C). Using a standard SBR formulation, the mechanical reinforcement decreased as the oxygen and hydrogen contents of these carbon blacks decreased (Table 4), but the reductions were not as severe as the literature results [7] for the heat treatment at the much higher temperature of 3000 °C (Table 1).

Early work on carbon black in the 1950s focused on the presence of free radicals on the surface of the carbon black particles [58]. It was shown by Kraus that the number of free radicals associated with carbon black is 10^19^ to 10^20^ spins/gram of carbon black [59,60]. Briefly, electron spin resonance (ESR) can be used to measure unpaired electrons in transition metal, organic, or any other specimen where unpaired electrons exist. The electron has associated with it two spin states of equal energy. In a strong magnetic field, the energies of the two spin states are split. Once the energy levels are split, radiation can be used to probe the absorption and emission in order to find the three main parameters of the ESR spectrum. The first item of interest is the g shift of the emission which is indicative of the chemical environment. The second item is the peak width which is indicative of localization and exchange processes of the unpaired electron, and the third parameter is the spins per gram that can be measured through the use of an internal standard [61]. 

**Figure 12:**
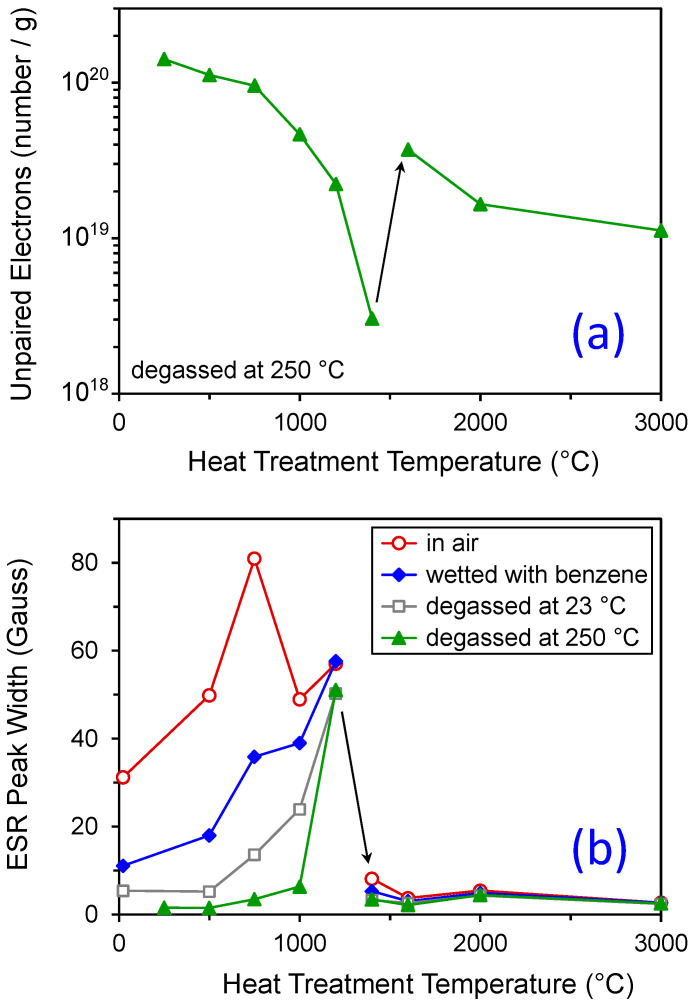
Electron spin resonance results ((**a**) unpaired electrons and (**b**) ESR peak width) for medium processing channel (MPC) type of carbon black (Spheron 6 from Cabot; NSA = 111.5 m^2^/g [64]). Results replotted from Kraus et al. [59,60].

According to Figure 12a [59,60], the number of unpaired electrons at the carbon black surface was decreased to a minimum when the carbon black was heat treated at 1400 °C. It was also noted that the line width was considerably reduced in the material that had been heat treated to above 1400 °C (Figure 12b). These two trends suggest that the first peak consists of delocalized pi electrons that share many different resonance structures, resulting in a broad line width ESR signal. The second peak is most likely due to localized sigma unpaired electrons that exist at one carbon atom and do not share/exchange interactions. The more localized signals could be in part due to CO and CO_2_ that are known to be evolved from the surface during the heat treatment process.

Interestingly, when an elastomer is masticated without carbon black, the free radicals that are created from polymer chain scission during mixing are very short lived. However, when carbon black is included in the mix, the free radicals are very long lived [62]. This is indicative that the carbon black is stabilizing the polymer free radical. The broken polymer fragment complex that is formed with the carbon black is extremely stable and would be referred to in ESR parlance as “trapped”. The species that are used to trap short lived free radicals form a stable adduct and that adduct itself is a free radical. The normal mechanism for the long-lived free radical is that the unpaired electron is able to delocalize, which would be reasonable given the large size of the graphene sheets and the readily available low energy gap conduction band of said graphene sheets. During mixing, it is commonly acknowledged that the carbon black aggregates undergo shear fracture and this in turn can also create free radicals. Herd and co-workers did a convincing job of showing the amount of carbon black fracture that typically occurs during mixing through careful TEM histogram study of rubber specimens after mixing [63]. The radicals generated by CB aggregate breakage could react with the polymer chains during compounding to form polymer–CB bonds, which may be more promoted in unsaturated elastomers such as NR and BR.

**Figure 13:**
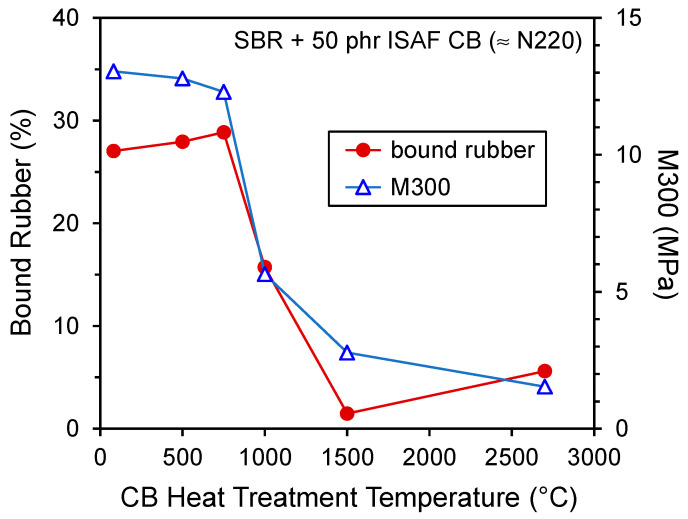
Bound rubber and M300 for SBR filled with ISAF CB (≈N220) versus CB heat treatment temperature. Results are replotted from Dannenberg [65].

Complex changes in carbon black surface chemistry occur with heat treatment. We showed that oxygen-containing functional groups disappear with annealing in the range from 900 to 1200 °C (Table 2). At the same time, there is a shift in the nature of free radicals that occurs at approximately 1400 °C (Figure 12). In terms of rubber properties as a function of CB heat treatment, Dannenberg [65] showed that both bound rubber and M300 (tensile stress at 300% strain) dropped significantly as a function of CB annealing temperature, and this transition occurred at approximately 1000 °C for an SBR formulation filled with N220 CB (see Figure 13). It is clear that the extreme temperatures often used to ‘graphitize’ carbon black (for example, 3000 °C for the literature results [7] in Table 1) are not necessary to remove the reinforcing effect of carbon black which occurs at much lower temperatures.

## 4. Processing Effects on Reinforcement

A solid preview of the importance of processing effects on CB reinforcement of elastomers was already given in the Introduction. CB aggregates can fracture during mixing [8,63,66] (see Figure 4) and bound rubber increases as mixing time is increased. These changes are correlated with enhancements in mechanical reinforcement (Figure 3). We will consider these observations further and discuss other processing-related considerations. 

It is generally noted that stress–strain properties of CB-filled rubber correlate better with COAN than OAN [67], suggesting that the high-pressure compression used in the COAN test for CB characterization mimics the CB structure breakdown during rubber mixing [68]. We already highlighted the mixing breakdown of CB aggregates in Figure 4, and this reduction in aggregate size during rubber compounding is generally greater for higher structure carbon blacks, stronger polymer–filler interactions, and more intensive mixing processes (longer time, higher torque) [8,69,70]. The COAN test method involves compressing a CB sample four times at a pressure of 165 MPa before performing the oil absorption experiment [71]. Not all CB aggregates are expected to be exposed to this high pressure, since it is known that jammed particle–particle force chains involving a fraction of the particles can shield stresses from the remainder of the particle population in granular materials [72,73]. Notwithstanding this, we can compare this COAN test pressure to typical values of pressure (*P*) and shear stress (*τ*) encountered in internal mixers and two-roll mills used in mixing rubber. This contrast is summarized in Table 5, where it is evident that the stresses are orders of magnitude smaller for the rubber processing operations [74,75,76,77,78]. Due to the shear-thinning nature of viscosity for polymer melts, it is difficult to generate shear stresses in excess of approximately 10 MPa in elastomers at processing temperatures, even when highly loaded with viscosity-boosting CB [79]. There are caveats to the simplistic comparison in Table 5. Due to the nearly incompressible nature of rubber, hydrostatic (volume) compression—which could occur in thin layers of polymer between filler particles in a rubber compound—can produce stresses in the GPa range. Additionally, the typical pressure and shear stress values for compounding of rubber in internal mixers and two-roll mills are continuum values that do not consider the nano- and micro-mechanics in the complex polymer–particle composite; local microscopic stresses could be significantly higher. 

Watson [80] proposed that elastomer chains undergo scission during rubber compounding processes to produce polymeric free radicals that subsequently react with CB surfaces to form chemically bound rubber. This work mainly focused on bound rubber results and did not show the corresponding stress–strain reinforcement effects. Convincing evidence for this mechanism included the observations of: (1) more bound rubber at lower mixing temperatures (higher shear stress breaks more polymer chains); (2) less bound rubber when free radical acceptors were included in the formulation; and (3) increased bound rubber when mixing was conducted in a nitrogen versus air environment due to radical-quenching activity of oxygen. An interesting and likely related finding is that bound rubber continues to increase after mixing during storage of uncured rubber compounds [81,82,83,84].

The influence of adding a second, intensive non-productive mixing stage on rubber properties was investigated for a variety of CB-filled tire tread compounds by Welsh and coworkers [85,86]. The carbon blacks studied were high structure grades that included N234 and N339, and the main elastomer system was an SBR/BR(70/30) blend. The intensive mixing procedure gave M300/M100 values that were increased 9 to 14% compared to the conventional mixing approach, with suggested origin from enhanced polymer–filler interactions developed during the intensive compounding conditions. Additional research studies that show rubber processing effects on dynamic mechanical behavior, tensile stress–strain behavior, and other physical properties are summarized by Wang [86].

**Figure 14:**
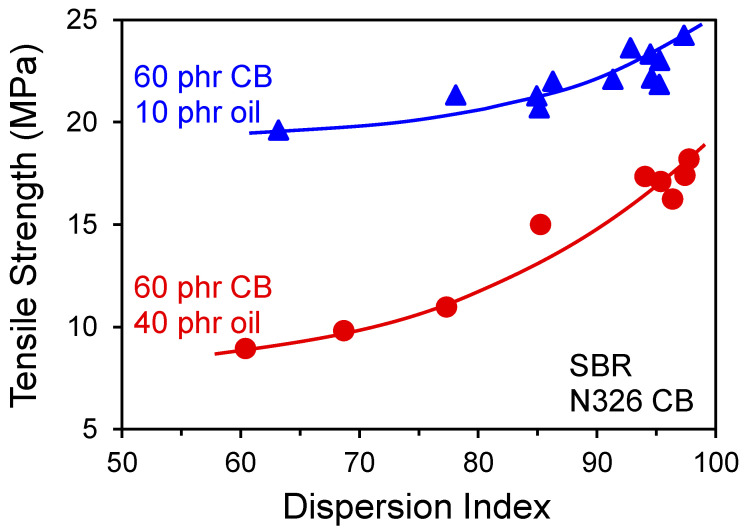
Effect of CB macrodispersion on tensile strength for filled SBR with the indicated loadings of CB and oil. Data replotted from Hess and coworkers [88].

The stress upturn zone of the tensile response of rubber (region 3 in Figure 8) is not significantly affected by minor amounts of micrometer-scale CB agglomerates from insufficient filler dispersion during mixing. However, failure properties are closely linked to such crack precursors/defects in rubber [40,87], hence poor filler macrodispersion causes reductions in tensile strength and elongation at break. The presence of large filler agglomerates essentially shifts the ultimate softening and break behavior (region 5 in Figure 8) to lower strains. Typical CB dispersion effects on tensile strength from Hess and coworkers [88] can be seen in Figure 14. It should be mentioned that the aforementioned CB aggregate fracture and polymer chain scission that can occur during compounding will both be further promoted by efforts to improve filler macrodispersion by increasing shear rate and mixing time, for example. 

## 5. Chemical Modification Effects

Mechanical reinforcement of rubber by carbon black is closely linked to the nature of the polymer–filler interface. There have accordingly been significant efforts across several decades aimed at improving the interactions between elastomer chains and carbon black by adding polymer–particle coupling agents to the rubber formulation, using functionalized elastomers, and modifying the CB surfaces. We will not give a comprehensive survey of the extensive industrial and academic research in this important area but rather offer some representative examples.

**Figure 15:**
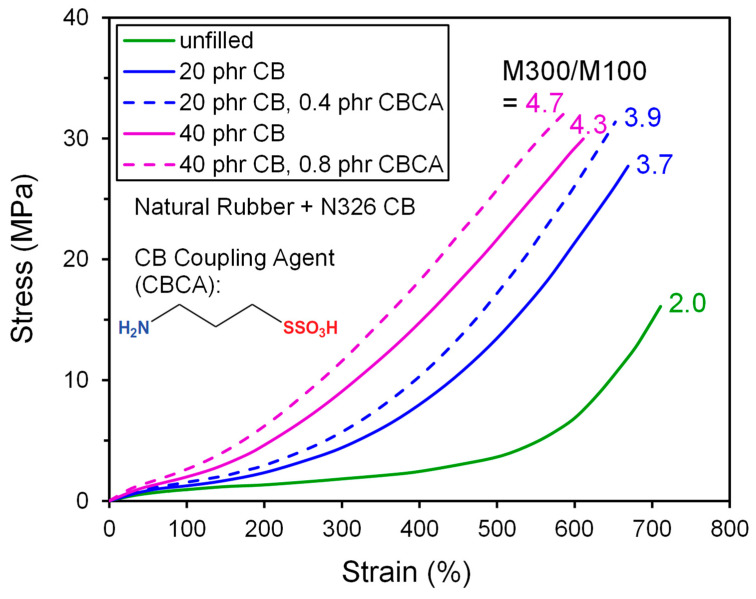
Tensile stress–strain results replotted from Han et al. [92] showing the reinforcement effects of adding the indicated coupling agent to N326 CB-filled NR formulations at two filler concentrations.

Coupling agents have been developed toward improving the interfacial bonding of elastomer chains and CB surfaces [89,90,91,92,93,94,95]. The various chemical groups on carbon black were already discussed (Figure 10c). This functionality is mainly on the edges of the graphene sheets, so the reactivity of CB is considerably less than precipitated silica which is entirely covered with -OH groups for potential reaction with silanes [36]. An example of the reinforcing impact of a carbon black coupling agent (CBCA) on the stress–strain curve for natural rubber is given in Figure 15 from results of Han and coworkers [92]. Also shown in this figure, the chemical structure of the key coupling agent used in this study has an amino group that reportedly reacts with carboxyl and aldehyde groups on the CB surface during mixing, and the other end bears a sulfur group for reacting with an unsaturated elastomer during the vulcanization stage. The stress–strain curves are steeper for the compounds with coupling agent added. The study also reported a modest increase in crosslink density from the CBCA due to the amino functionality apparently acting to accelerate sulfur vulcanization. This cure influence makes it difficult to assign responsibility for the improvement in reinforcement to a simple polymer–filler coupling phenomenon. 

Traditional silane coupling agents that are used in rubber reinforced with precipitated silica, such as the common bis(triethoxysilylpropyl)tetrasulfide (TESPT), also show come coupling activity when the filler is carbon black [96,97] due to presumed reaction with the small amount of hydroxyl groups on CB. The bound rubber and mechanical reinforcement effects that are noted in CB-filled rubber are much smaller than observed for silica compounds, and the S4 in TESPT is known to increase the crosslink density which confuses the situation.

Raut and coworkers [91] demonstrated that pi-pi interactions with the graphene on carbon black can provide a route for physically coupling elastomers to CB surfaces. Their coupling agent—formed from grafting polypentafluorostyrene onto polybutadiene—provided enhanced polymer–CB interactions in N234 CB-reinforced SBR compounds as verified by substantial reduction in filler networking (lower Payne effect) but with reduced reinforcement in the larger strain region (decreased M300/M100). 

Polybutadienes and styrene–butadiene rubbers produced by anionic polymerization (solution process) can be chain-end functionalized for reaction with carbon black, precipitated silica, and other fillers during mixing for improved performance of tire treads and other applications [98,99,100]. For carbon black, the tin (Sn) functionality is well known for developing elastomer–CB bonds [101,102,103]. Model reaction studies were performed by Tsutsumi et al. [102] to test the reactivity of the assorted functional groups that are found on CB surfaces (Figure 10c) with Sn-coupled low molecular weight polybutadiene, and clear reactivity was found with β-naphthoquinone which provided support for Sn-functionalized elastomers reacting with the quinone groups on CB in rubber compounds to form covalent polymer–CB bonds. The research on Sn-functionalized elastomers is often focused on the influence of the modified polymers on small strain dynamic mechanical properties [103,104], but the large strain filler reinforcement is also affected. The tensile properties for SBR terminated with trioctyl tin chloride are compared to its non-functionalized counterpart in Table 6 from an investigation by Escobar Barrios and Garcia-Ramirez [101] for N330 CB-filled compounds. The Sn functionality led to a steeper stress upturn in the tensile curve (greater M300/M100 ratio). The curing time was approximately 10% slower for the Sn-terminated SBR in this study, so the crosslinking kinetics were not greatly affected by the chain-end functional group.

The reduction in CB surface functionality due to annealing at approximately 1000 °C was described previously. Surface oxidation of carbon black using acid, ozone, and oxygen plasma treatments has the opposite effect, and the enhanced surface activity increases bound rubber and gives moderate enhancements to reinforcement in filled compounds with various elastomers [105,106,107,108]. Other surface modification approaches—including grafting polymers onto CB—are reviewed elsewhere [109,110,111].

## 6. Influences of Carbon Black on Crosslinking

Cure kinetics for sulfur vulcanization of elastomers speed up significantly when carbon black is present in the formulation [9,10], and any related CB-induced changes to crosslink density or spatial distribution of crosslinks may contribute to the mechanical reinforcement. This cure acceleration from CB was already demonstrated in Figure 5 for CB-filled SBR [9], and many other examples can be found in the scientific literature, such as faster scorch and shorter optimum cure time for nitrile butadiene rubber (NBR) from addition of N330 CB [112]. Brennan and Lambert [113] reported increased crosslink density for CB-reinforced BR compounds compared to unfilled polybutadiene, and the extent of crosslinking increase was proportional to the bound rubber which was elevated either from increased CB concentration or use of CB grades with higher surface area. Their study also showed small increases in bound rubber after annealing at curing temperatures for compounds without curatives in the formulation.

Blokh and Melamed [114] demonstrated direct reaction of carbon black with sulfur when heated at 145 °C, and continuous extraction with benzene for 25 days could not separate the bound sulfur from the CB. Reactivity was also shown between CB and common accelerators used in rubber formulations, mercaptobenzothiazole (MBT) and tetramethylthiuram disulfide (TMTD). These researchers also pre-reacted CB with sulfur and accelerators and added the mixture to rubber compounds, which produced increased tensile strengths in NR, BR, and NBR formulations compared to conventional rubber compounds with all ingredients added individually during mixing. 

Mechanical testing and swelling measurements were used by Gent, Hartwell, and Lee [115] to study the influence of N330 CB on crosslinking of BR and isoprene rubber (IR). The swelling of CB-filled rubber crosslinked with various levels of curatives was proportional to the unfilled rubber swelling, and this was interpreted as evidence that crosslink density was not affected by the carbon black. The reduced extent of swelling from the presence of carbon black was attributed to “bonding of rubber molecules to, and between, filler particles”. These elastomer–CB bonds were strong enough to withstand solvent swelling stresses and heating up to 120 °C. Such strong attachments of polymer to CB were also evident in the NMR and swelling research of Valentín et al. [116] which concluded that some fraction of the elastomer is connected to the CB surface and acts as a giant crosslink, but the nature of crosslinking within the bulk rubber is otherwise not significantly affected.

The rate of crosslinking for NR and SBR compounds containing regular, heat-treated, or oxidized carbon blacks was found to be inversely proportional to the CB surface acid concentration [117]. This suggests that the CB surface functionality is important for cure effects. However, the graphene chemical structure—which makes up the majority of the carbon black surface (Figure 10)—may also influence the crosslinking process. Wu et al. [118] showed that adding graphene as a reinforcing filler to natural rubber caused increases in crosslink density as determined from swelling measurements and torque increases in a cure rheometer as well as faster cure kinetics from differential scanning calorimetry (DSC) and rheometry. In another study [119], the graphene surfaces of single-wall carbon nanotubes (SWCNTs) gave a cure acceleration effect similar to carbon black in NR formulations. 

Cabot Corporation [120] showed that rubber-to-CB crosslinks can increase degree of reinforcement. They developed an approach to functionalize the surfaces of previously heat-treated carbon blacks with disulfides. The heat treatment process they used to reduce polycyclic aromatic hydrocarbon (PAH) content in the CBs had a negative effect on rubber reinforcement, comparable to the CB heat treatment effect we already introduced (Figure 2, Table 1 and Table 2). The disulfides can participate in the sulfur vulcanization to create chemical linkages between CB surfaces and elastomer chains, and this was found to partially counter the loss of reinforcement for SBR/BR and natural rubber (NR) compounds (Table 7). 

## 7. Possible Polymer–Filler Interactions

**Figure 16:**
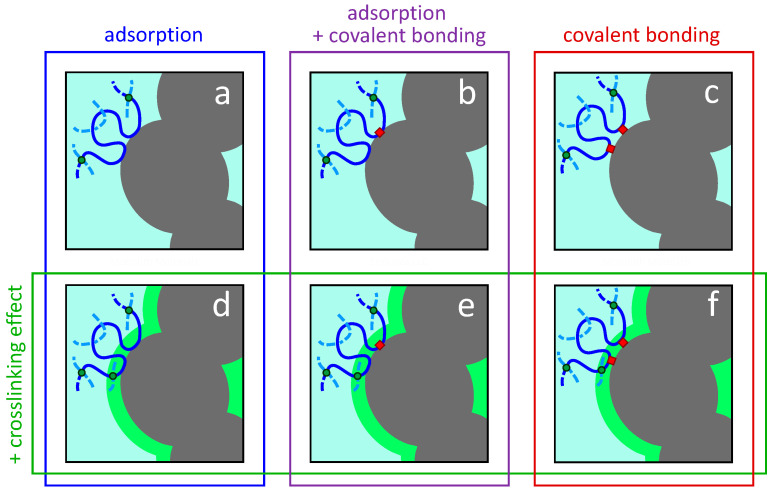
Possible polymer–filler interaction/reaction scenarios for crosslinked elastomer reinforced with nano-structured filler aggregates. A portion of filler aggregate is illustrated (gray) with one full primary particle shown that is fused to two other primary particles. The solid blue line represents a polymer network chain between crosslinks (green dots). Covalent polymer–filler bonds are indicated with red squares. The green halo surrounding the filler aggregate in (**d**–**f**) signifies a region of increased crosslink density due to proximity to filler, with an additional crosslink (green dot) therein compared to (**a**–**c**). A discussion of relative sizes of polymer network chains and primary particle diameters for CB-filled rubber is given elsewhere [122].

We present the possible polymer–filler interaction and reaction scenarios for a crosslinked elastomer reinforced with particles in Figure 16. If the CB type is a high surface area grade such as N110, N115, or N134, and the crosslink density is in a normal range for industrial rubber compounds [121], then we can assign approximate dimensions of 15 nm × 15 nm to each box in this figure. A recent paper discusses the relative sizes of polymer network chain between crosslinks and the carbon black particles [122]. The primary particles and aggregates increase in size for grades with lower surface areas (Table 3), dwarfing the average distance between crosslinks when N550, N660, and N762 are considered. 

For general-purpose elastomers such as polybutadiene, EPDM, butyl rubber, NR, and SBR, it is widely accepted that elastomer chains are adsorbed onto CB surfaces to an appreciable degree. Given the free radical chemistry that can occur between polymer chains and CB aggregates during mixing, covalent polymer–particle linkages are also probable, although likely less extensive than the adsorption sites (Figure 16b). Although not relevant for typical carbon blacks with common elastomers, it is possible to have chemical bonds between polymer and filler without chain adsorption (Figure 16c) such as in silica-silane-rubber systems. 

The presence of CB in a rubber formulation accelerates the sulfur vulcanization kinetics compared to unfilled elastomers. Consequently, it is a reasonable proposal that the crosslink density near the filler surfaces is elevated compared to the bulk rubber, and this is depicted in the lower half of Figure 16 (scenarios d, e, and f). This hypothetical layer would have elevated modulus to help bridge the GPa-to-MPa modulus gap between rigid particle and rubbery polymer, with positive implications on both material stiffness and strength.

Bound rubber [123,124,125,126] measurements have limited applicability in distinguishing the polymer–filler interaction/reaction effects illustrated in Figure 16, although more insights are offered when the bound rubber is rigorously separated into physical and chemical components [127]. We would like to know the polymer–filler interaction/reaction situation in the final cured state, but bound rubber can only be used for investigating the uncured mixed compound. Covalent bonds between polymer and CB can continue to form during high temperature annealing in the curing process, and the carbon black can influence and/or participate in the crosslinking. Advanced microscopy [128,129] and NMR [116,130] techniques can offer important insights but are not conclusive and cannot distinguish a strong polymer adsorption on the filler surface from a chemical bond. If the research results covered in this review are considered as a whole, we believe that the most realistic case is adsorption + covalent bonding + crosslinking effect (scenario e), but this is difficult to conclusively prove experimentally for cured rubber.

## 8. Reinforcement Models

In this final section, we briefly discuss various reinforcement models that have been proposed. Some of these are not predictive mathematical models that can be used to fit and interpret the stress–strain behavior of filled rubber but rather are conceptual frameworks for rationalizing reinforcement from carbon black. The challenge for any theory of CB reinforcement of elastomers is to explain the stress upturn enhancement from filler while also capturing softening behavior such as the well-known Mullins effect [131,132].

Early reinforcement ideas included a general framework of coexisting strong and weak bonds between polymer and carbon black by Blanchard and Parkinson [133] and Bueche [134]. Dannenberg introduced a molecular slippage and stress redistribution concept in an attempt to explain the complex reinforcing effect of carbon black in rubber, and this concept relies entirely on physical interactions of elastomers adsorbed on CB surfaces [65,135]. These general mechanistic notions were not developed into predictive expressions.

Consistent with the idea of interfacial slippage, Rigbi [136,137] developed a model based on elastomer chain detachments and reattachments (“saltations”) to carbon black surfaces using rate theory of liquid viscosity [138] in combination with polymer chain statistics and rubber elasticity theory. Time-dependent stress relaxation, creep behavior, and the rate- and temperature-dependent stress–strain curve can be generally predicted. However, the Rigbi model cannot simultaneously explain CB-induced increases in both stiffness and strength. In an attempt to resolve this major deficiency, the author proposed that weak polymer–filler interactions are needed for strength—which is contrary to the chemical modification efforts mentioned earlier—but at the same time there needs to be many such weak polymer–particle adsorptions to yield increased modulus.

Fukahori [139] proposed a carbon black reinforcement model based on glassy hard (GH) and sticky hard (SH) elastomer layers. The GH layer was justified based on dynamic mechanical measurements on CB–polymer gel (bound rubber and CB remaining after solvent extraction and drying in a bound rubber test) that showed that the bound rubber is at least two times stiffer than the bulk rubber. This enhanced stiffness of rubber near CB surfaces has also been noted by atomic force microscopy [129,140]. Each SH layer filament interconnects two GH layers around CB particles. Molecular slippage within polymer entanglements and related stress redistribution occur within the SH regions, and the GH layer limits the amount of slipping possible in the SH layer. This general conceptual framework leads to a super-network of strands of oriented molecules at large strain that interconnect the carbon black aggregates and can theoretically produce the stress upturn that is observed experimentally for CB-reinforced rubber. No distinction of covalent versus physical polymer–filler bonds is made concerning the glassy hard layer, but that region is considered to be tightly bound to the carbon black. 

Witten, Rubinstein, and Colby developed a scaling theory to explain the stress–strain upturn behavior for rubber reinforced with branched filler aggregates such as CB particles [141]. According to their model, uniaxial extension of rubber filled with fractal filler aggregates results in a lateral compression of the filler aggregates, driven by the large bulk modulus of the nearly incompressible elastomer. The bulk modulus of rubbery polymers and relative values of Poisson’s ratio for polymer and filler network are also important for small strain reinforcement in the Payne effect regime [142]. Strong bonding between elastomer and filler particles is implied by the Witten–Rubinstein–Colby scaling model, because weak interfaces would otherwise allow polymer–particle debonding and cavitation to alleviate the stresses that produce this proposed filler reinforcement effect. 

A dynamic flocculation model was developed by Klüppel and coworkers [8,143,144] and successfully implemented to fit experimental stress–strain cycles involving histories with varied amplitudes and different deformation modes for various elastomers reinforced with carbon black, precipitated silica, and other fillers. The approach is based on the non-affine tube model of rubber elasticity for the elastomer matrix which is reinforced via strain-amplification from filler aggregates. The possibility of breaking some filler cluster-cluster bonds during deformation introduces a softening behavior for capturing the Mullins effect. A glass-like polymer layer around the filler particles—that can soften with deformation and reduce in thickness with increasing temperature—is proposed to be the region where breaking and reforming of bonds between fractal filler clusters occurs. They are termed cluster-cluster bonds or filler-filler bonds, but the polymer layer is the alleged “glue” such that they are really filler-polymer–filler bonds. However, the continuum model has hypothetical weak and strong bonds between filler clusters and does not include any real microstructural details of the polymer–filler interfacial region. The model supports the idea of strong polymer–particle bonding in a few ways. The notion that there is a glassy layer of polymer at the surface of the filler requires a high degree of interaction between the elastomer chains and filler. Comparing the fitting results for unfilled elastomers with elastomers filled with carbon black, the latter exhibit a higher value of G_c_ which is the modulus parameter that is proportional to crosslink density. The model fitting accordingly indicates a higher crosslink density coming from the addition of CB which we suggest could be from direct covalent linkages (Figure 16b) or enhanced crosslink density near the filler surfaces (Figure 16d). Klüppel goes through a lengthy examination in a review chapter [8] to highlight the possibility of strong physical adsorptions for diene elastomers on carbon black surfaces, because strong polymer–filler bonds are indeed presumed by the model. The bonds in the model are mathematical constructs, and the model cannot discriminate between covalent elastomer–CB bonds versus strong physical interactions. The ideas in the dynamic flocculation model have been extended and improved by Plagge et al. [145,146] into a mathematically-robust constitutive modeling approach that can be implemented in finite element analysis.

## 9. Final Comments

Carbon black is a highly engineered nano-structured filler, and the mechanical performance of CB-filled rubber is a complex research area that is still being studied and debated today despite nearly 100 years of prior study. We reviewed the rich structure and chemistry of carbon black particles toward a better understanding of their widely successful reinforcement of rubber. The surface area, aggregate structure, and surface chemistry all contribute to the reinforcing nature of carbon black when used as a filler in elastomers. Heat treatment of CB at approximately 1000 °C removes surface functional groups without significantly affecting the surface area, aggregate shape, or graphitic structure, and this greatly reduces the bound rubber and mechanical reinforcement. Considering the research literature in total, we believe that the most realistic scenario for polymer–CB interfaces is predominantly physical adsorption with some covalent chemical bonds also present. The relative amount of physical interactions is presumably 10–100x in terms of number of interaction points compared to chemical bonds. However, the covalent bonds are so strong compared to van der Waals interactions that, even if there are 30 physical interactions for every one covalent bond, the total amount of bond enthalpy would be approximately equal. The adsorbed chain segments can slide/detach/re-attach to redistribute stresses and yield Mullins softening, and the covalent polymer–particle linkages enhance the stress upturn in tensile deformation and resist interfacial debonding. Additional complexities include aggregate breakage and free radical chemistry during compounding as well as the accelerating effect of CB on the sulfur vulcanization of rubber. The latter may produce a layer around the particles with increased crosslink density compared to the bulk elastomer. However, additional analytical research is needed since the exact nature of the polymer–filler interfaces in the final cured rubber has yet to be conclusively diagnosed. 

## Figures and Tables

**Table 1:** Effects of Heat Treatment of CB on Properties of Styrene–Butadiene Rubber (SBR).

	High Structure ISAF CB (≈N234)		ISAF CB (≈N220)	
Property	Original	Heat Treated ^(a)^	Original	Heat Treated ^(a)^
CB; Nitrogen Surface Area (m^2^/g)	116	86	108	88
CB; Oil Absorption (cm^3^/g)	1.72	1.78	1.33	1.54
Bound Rubber (%)	34.4	5.6	30.6	5.8
Mooney Viscosity at 100 °C	83	87	73	76
Scorch at 135 °C (min.)	10.5	17	18	20
Filler Macrodispersion (%)	99	99	99	98.2
Shore A Hardness	73	68	68	65
Stress at 300% Strain, M300 (MPa)	14.5	3.5	10.3	2.9
Tensile Strength (MPa)	26.2	23.4	27.6	22.8
Strain (Elongation) at Break (%)	450	730	630	750
Abrasion Loss (cm^3^/10^6^ rev.)	62	181	67	142
Hysteresis	0.204	0.297	0.238	0.315

Data from Brennan, Jermyn, and Boonstra [7]; ^(a)^ CB annealed at 3000 °C for 1 h; ISAF: intermediate super abrasion furnace.

**Table 2:** Effects of Heat Treatment on CB Characteristics.

CB Sample	NSA (m^2^/g)	STSA (m^2^/g)	O (%)	N (%)	H (%)	S (%)	C (%)	L_c_ (nm)	d_002_ (nm)
N234, untreated	126.4	120.3	2.21	0.145	0.337	0.924	93.7	1.19	0.365
N234, 900 °C	134.7	124.7	1.28	0.158	0.250	0.932	95.9	1.15	0.361
N234, 1000 °C	129.6	129.6	0.204	0.064	0.130	0.916	96.7	1.40	0.361
N234, 1200 °C	129.0	132.8	0.128	0.041	0.021	0.790	98.7	1.44	0.355
N660, untreated	36.4	35.2	0.576	0.082	0.339	1.84	95.9	1.78	0.352
N660, 1000 °C	36.4	37.3	0.110	0.056	0.141	1.78	96.8	1.59	0.355

Data from Monolith Technical Center in Lincoln, NE. Heat treatment conditions: CB annealed in inert atmosphere under positive-pressure Ar flow at indicated temperature for 18 h. Elemental analysis results from Leco ONH836 and Leco SC832 Elemental Analyzers. Crystallographic data (L_c_ and d_002_) from Rigaku Miniflex powder X-ray diffractometer utilizing k alpha radiation.

**Table 3:** Surface Area and Structure Characteristics of Various Furnace Grades of CB for Rubber.

Carbon Black Grade	NSA (m^2^/g)	STSA (m^2^/g)	OAN (ml/100 g)	COAN (ml/100 g)	Mean Aggregate Diameter (nm) ^(a)^
N115	131	116	112	93	64
N134	140	129	125	104	63
N220	110	103	113	99	78
N234	116	110	126	104	67
N339	91	88	121	99	76
N330	76	76	102	89	84
N326	77	77	73	73	82
N550	38	38	121	83	179
N660	35	34	93	75	168
N772	32	31	69	62	169

^(a)^ Size of CB aggregates tested by disk centrifuge photosedimentometry after compressing the CB four times at 165 MPa (compressing/crushing identical to the COAN test). Data from Tunnicliffe [47].

**Table 4:** Effects of CB Heat Treatment on Tensile Properties of CB-Filled SBR.

CB Sample	M100 (MPa)	M300 (MPa)	M300/M100	Tensile Strength (MPa)	Elongation at Break (%)
N234, untreated	2.68	15.33	5.72	17.9	361
N234, 900 °C	2.77	15.33	5.54	19.1	375
N234, 1000 °C	2.11	11.11	5.27	22.8	493
N234, 1200 °C	1.78	7.78	4.36	21.4	560
N660, untreated	2.59	13.33	5.15	18.9	429
N660, 1000 °C	1.96	7.99	4.08	15.2	516

Data from Monolith Technical Center in Lincoln, NE (see Table 2 for CB characteristics). Heat treatment conditions: CB annealed in inert atmosphere under positive-pressure Ar flow at indicated temperature for 18 h. Results from room temperature tensile testing for emulsion SBR rubber formulation specified in ASTM D 3191.

**Table 5:** Pressure in the COAN Test Compared to Pressures and Stresses in Rubber Processing Equipment.

Compression in the COAN Test	Rubber in Internal Mixer [74,75,76]		Rubber in Two-Roll Mill [77,78]	
*P* (MPa)	*P* (MPa)	*τ* (MPa)	*P* (MPa)	*τ* (MPa)
165	0.2–0.7	0.3–7	0.2–3	0.2–1

**Table 6:** Effects of Tin Chain-End functionalization of SBR in CB-Filled Rubber [101].

	SBR Non-Functionalized	SBR Functionalized with R_3_SnCl *
M100 (MPa)	4.03	4.01
M300 (MPa)	15.26	17.02
M300/M100	3.79	4.24
Tensile Strength (MPa)	17.83	17.73
Elongation at Break (%)	378	346

* Anionically-polymerized (solution) SBR terminated with trioctyl tin chloride.

**Table 7:** Disulfide Functionalization of CB to Counter Reinforcement Loss from Heat Treatment.

	SBR/BR Compound (*ϕ* = 0.22)		NR Compound (*ϕ* = 0.19)	
Carbon Black	tanδ at 60 °C	M300/M100	tanδ at 60 °C	M300/M100
CB1; Control, STSA = 75 m^2^/g, OAN = 102	0.257	4.12	0.169	5.31
CB2; CB1 annealed for 2 h at 1400 °C	0.329	2.20	0.192	3.16
CB3; CB2 surface funct. with disulfide	0.255	3.00	0.135	4.29

Data from U.S. Patent granted to Cabot Corporation [120].

## Data Availability

Not applicable.
